# Predictive model establishment for forward-head posture disorder in primary-school-aged children based on multiple machine learning algorithms

**DOI:** 10.3389/fbioe.2025.1607419

**Published:** 2025-05-30

**Authors:** Hongjun Tao, Yang Wen, Rongfang Yu, Yining Xu, Fangliang Yu

**Affiliations:** ^1^ Department of Physical and Education, Anhui Jianzhu University, Hefei, China; ^2^ College of Sports Industry and Leisure, Nanjing Sport Institute, Nanjing, China; ^3^ Ningbo New Fitness Health Technology Co., Ltd., Ningbo, China; ^4^ Faculty of Sports Science, Ningbo University, Ningbo, China; ^5^ School of Sport Training, Nanjing Sport Institute, Nanjing, China

**Keywords:** primary school-aged children, forward head posture, machine learning, risk prediction model, shapley additive explanation algorithm

## Abstract

**Background:**

Forward head posture frequently occurs among primary school children, potentially due to prolonged sedentary behavior associated with academic demands and reduced physical activity. However, existing prevention and screening methods fail to accurately and promptly predict the onset of forward head posture.

**Objective:**

This study aims to identify highly sensitive predictive indicators for forward head posture in primary school children using the Least Absolute Shrinkage and Selection Operator (LASSO) regression algorithm. Multiple machine learning algorithms are applied to construct distinct risk prediction models, with the most effective model selected through comparative analysis. The Shapley Additive Explanations (SHAP) method is used to quantify the influence of each feature on model outcomes, ensuring enhanced model interpretability.

**Methods:**

Employing a cross-sectional study design, this research recruited 520 primary school-aged children, gathering data on demographics, anthropometrics, and physical activity levels. Univariate logistic regression was utilized to identify high-risk factors for forward head posture. The LASSO algorithm was subsequently applied to select key predictors. Six machine learning models—K-nearest neighbor (KNN), light gradient boosting machine (LGBM), extreme gradient boosting (XGBoost), random forest (RF), linear model (LM), and support vector machine (SVM)—were developed to predict risk. The performance of each model was evaluated, and the best-performing model was further interpreted using the Shapley Additive Explanations (SHAP) algorithm.

**Results:**

A total of 514 children were ultimately included in the study, of whom 300 exhibited forward head posture. LASSO analysis identified age, bodyweight, BMI, sex, and weekly total homework time as prominent risk indicators. Among the 6 predictive models, the random forest algorithm demonstrated the highest performance (AUC = 0.865), significantly outperforming the others. SHAP analysis revealed that BMI, bodyweight, and age were the most influential predictors, with BMI contributing the most.

**Conclusion:**

The random forest-based prediction model achieved superior predictive accuracy for forward head posture among Chinese primary school children, emphasizing the importance of monitoring BMI, bodyweight, and age for early intervention and prevention efforts.

## 1 Introduction

As the primary sensory input center of the human body, the head’s posture plays a crucial role in maintaining overall spinal health (Sikka et al., 2020). Forward head posture is one of the most prevalent postural disorders among primary school-aged children and is typically diagnosed by measuring the cervical vertebrae angle (CVA) (Chu et al., 2020). Although a definitive threshold distinguishing normal from abnormal CVA remains debated, clinical consensus generally considers a CVA of less than 50° in a standing position indicative of forward head posture (Aoyama et al., 2020). A cross-sectional study published in 2016 reported an incidence rate of 53.5% among school-aged children, with particularly high prevalence in the 13–15 age group (SIngh et al., 2020). Furthermore, a 2022 study found that individuals with prolonged electronic device usage also exhibited a higher prevalence of forward head posture (Arooj et al., 2022).

For primary-school-aged children, the development of forward head posture is associated with multiple factors, including age, height, bodyweight, body composition, muscular strength, and growth rate (Arooj et al., 2022). During growth spurts, the increase in skeletal muscle mass and strength may fail to keep pace with the rapid growth of bones, resulting in imbalanced development between the musculoskeletal systems and leading to abnormal head and neck posture (Martinez-Merinero et al., 2020). Additionally, unhealthy habits—such as prolonged sedentary behavior and extended use of electronic devices—can cause compensatory changes in spinal curvature. These postural adaptations may result in overuse and stiffness of certain muscle groups, while others become relatively lax due to disuse, ultimately disrupting head posture and contributing to chronic pain and functional motor impairments (Rani et al., 2023; Suwaidi et al., 2023). Improper desk and chair heights may further induce maladaptive changes throughout the spinal and shoulder joint complex, prompting compensatory muscular responses that negatively affect cervical alignment and head posture. These biomechanical disruptions may trigger cascading postural issues such as kyphosis and posterior pelvic tilt (Martinez-Merinero et al., 2020; Rani et al., 2023). Lastly, children’s physical activity levels and visual acuity are also linked to the incidence of forward head posture. A sedentary lifestyle can diminish muscular strength and flexibility, while poor ergonomic setups and prolonged screen use may contribute to vision deterioration, collectively heightening the risk (Rani et al., 2023; Suwaidi et al., 2023). Therefore, the onset of forward head posture in primary school children is a multifactorial process involving biomechanics, lifestyle patterns, and physical activity.

In recent years, the widespread availability of medical big data has facilitated the extensive application of machine learning (ML) in risk prediction, offering more accurate approaches for identifying postural abnormalities in primary school-aged children. For instance, scoliosis prediction algorithms based on ML analyze large-scale biomechanical data and imaging information to enhance the precision of early diagnosis (Roland et al., 2016; Yong et al., 2018). Moreover, ML techniques have been employed in gait analysis among children to detect and forecast abnormal gait patterns, thereby informing the development of targeted corrective interventions (Prakash et al., 2016; Figueiredo et al., 2018; Khera and Kumar, 2020). To address the “black box” nature of ML models, the Shapley Additive Explanation (SHAP) algorithm has been introduced. This method quantifies the contribution of each clinical feature to the model’s output, providing transparent interpretability (Nohara et al., 2022), which aids pediatric healthcare providers, parents, and schools in understanding the causal relationships behind model predictions and formulating preventive strategies accordingly. Beyond model development and validation, evaluating the clinical utility of these models is essential to ensure the effectiveness and practicality of the recommended measures, thereby reinforcing their scientific validity and efficiency in real-world applications.

This study aims to identify highly sensitive predictive indicators for forward head posture in primary school children using the Least Absolute Shrinkage and Selection Operator (LASSO) regression algorithm. Multiple machine learning algorithms are applied to construct distinct risk prediction models, with the most effective model selected through comparative analysis. The Shapley Additive Explanations (SHAP) method is used to quantify the influence of each feature on model outcomes, ensuring enhanced model interpretability.

## 2 Methods and materials

### 2.1 Participants recruitment and screening

This study used a cross-sectional design. On 8 March 2025, 12 public primary schools in Nanjing, Jiangsu Province, China were randomly chosen (one from each of the city’s 12 districts). One class from each grade in each school was randomly picked. A notice was sent out, and legal guardians signed up voluntarily. Student data was collected on 9 March 2025.

Inclusion criteria: (1) Children aged 6–12 years; (2) Legal guardians signed up to participate voluntarily and provided informed consent, permitting the use of data for statistical analysis; (3) Absence of clinical contraindications to physical activity, including but not limited to genetic disorders and congenital disabilities; (4) Ability to comprehend basic instructions, remain conscious, and complete assessments under the guidance of testing personnel.

Exclusion criteria: (1) Presence of physical impairments or mobility limitations; (2) Congenital organic hearing or speech impairments that prevent test participation; (3) Any clinical contraindication to physical activity; (4) Diagnosed neurodevelopmental disorders, schizophrenia spectrum, or other psychotic disorders that would prevent full cooperation during assessments, as defined by the Diagnostic and Statistical Manual of Mental Disorders, Fifth Edition (DSM-5) (Regier et al., 2013).

**FIGURE 1:**
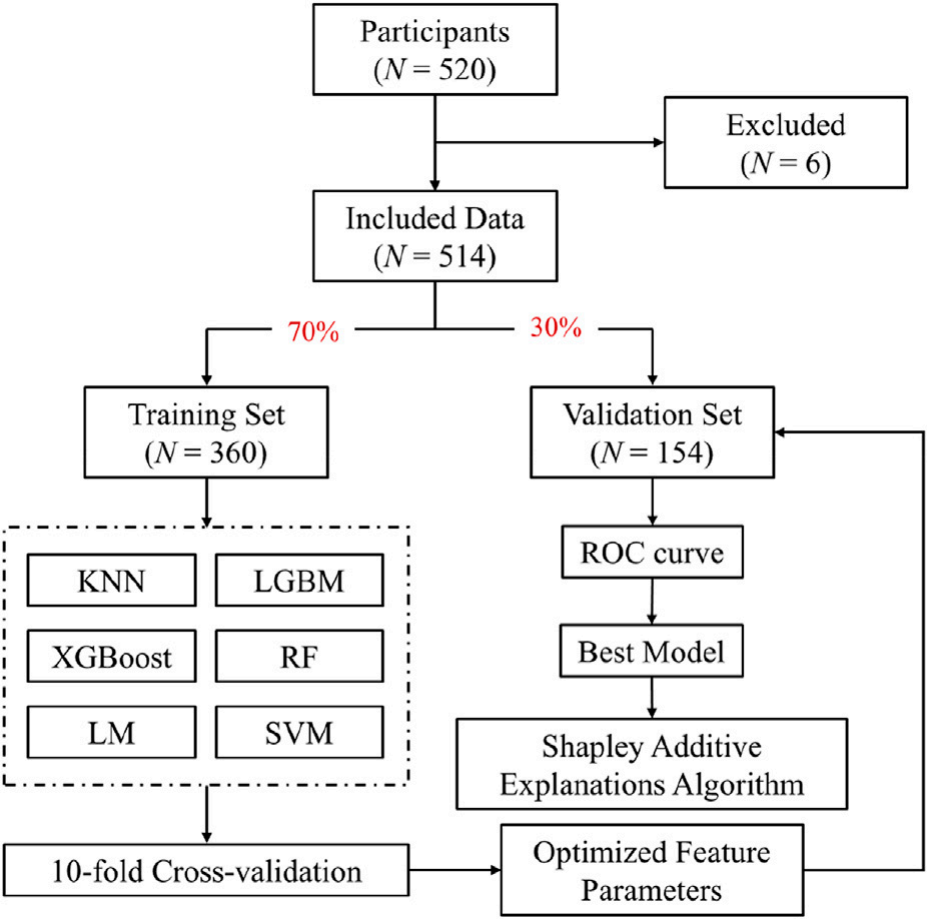
Flow diagram of the study.

Figure 1 is the flow diagram of the study.

### 2.2 Data collection

Data measurement and collection for this study were jointly conducted by certified testers and data screeners from the Department of Physical and Education, Anhui Jianzhu University. Comprehensive details regarding data collection procedures are provided in Table 1. The study received ethical approval from the Ethics Committee of Anhui Jianzhu University (AJU202400035), and informed consent was obtained from all participants’ legal guardians. Data definition and materials collection approach of the study was listed in Table 1.

**TABLE 1:** Data definition and materials collection approach of the study.

Item	Definition	Collection
Name	—	Questionnaire
Sex	—	Questionnaire
Age (year)	—	Questionnaire
Medical history	—	Questionnaire
Duration of seated study time outside of school hours		Questionnaire
Physical Activity	—	Questionnaire
Height (cm)	—	Questionnaire
Bodyweight (kg)	—	Inbody
BMI (kg/m^2^)	BMI = bodyweight(kg)/height^2^(m^2^)	Calculate
Body-type	Obesity/Overweight/Standard/Thin/Very thin	WHO standard (Wang and Wang, 2000)
Cervical vertebrae angle (°)	Angle formed between the line connecting the external auditory canal and the C7 vertebral prominence landmark and the horizontal plane	Kinect
Ear-to-shoulder distance (cm)	Horizontal distance between the ear canal and the shoulder	Kinect
Horizontal angle of shoulders (°)	The angle between the line connecting the left and right shoulder points and the horizontal plane	Kinect

**FIGURE 2:**
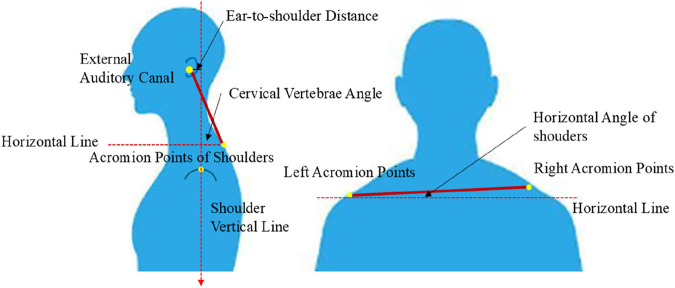
Scheme of human body posture recognition and relative data collection.

This study used a Kinect posture recognition and measurement device (Version: Kinect 2 for Windows 2014; Microsoft, USA) based on a depth camera to collect participants’ anthropometric data. This device uses depth camera technology and has an accuracy of 0.001 m. During the collection of the cervical vertebrae angle, ear-to-shoulder distance, and bilateral acromion horizontal angles, participants were instructed to face the Kinect depth camera directly, standing at a designated distance of 2 m from the lens. While maintaining a static anatomical standing position for 5 s, the device captured frontal posture data. Participants then turned 90° clockwise to record left-side static posture, followed by another 5-s anatomical stance. Subsequently, they turned 180° clockwise to capture right-side static posture, again holding the anatomical position for 5 s. This sequence concluded the posture data collection process. The process of posture recognition and associated data acquisition is showed in Figure 2.

Demographic and health-related information, including name, age, sex, exercise habits, and medical history of each participant, was collected through a structured questionnaire. Anthropometric indicators comprised height, bodyweight, and body mass index (BMI, unit: kg/m^2^). Height and bodyweight were measured using an electronic height–bodyweight measuring device, following standardized procedures. The height device had a measurement range of 60–200 cm with a precision of 0.1 cm, while the bodyweight scale operated within a 30–200 kg range with an accuracy of 0.1 kg. Body composition, including BMI, lean body mass, and muscle mass, was assessed using the InBody bioelectrical impedance body composition analyzer (Model 370, InBody Co., South Korea). During measurement, participants were required to remove hats and sports shoes and wear light clothing. All measurements were recorded to one decimal place to ensure accuracy. Lean body mass (kg) was calculated by subtracting fat mass (kg) from total body bodyweight (kg). The BMI formula is shown in Equation 1.

$$\text{BMI }\left(\text{kg}/m^{2}\right)=\text{Bodyweight }\left(\text{kg}\right)\;/\;{\text{Height}}^{2}\;\left(m^{2}\right)$$ (1)

Physical activity data was collected using the Chinese version of the Physical Activity Questionnaire for Adolescents (PAQ-A). This is a localized version of the international PAQ-A scale, designed to assess physical activity levels in adolescents. It was first developed by Canadian researcher Kowalski and later translated and revised by Chinese scholars to fit the habits and culture of Chinese adolescents. The Chinese version of the PAQ-A consists of 9 items that assess various dimensions of physical activity, including school physical education classes, extracurricular exercise, daily routines such as walking and household chores, and weekend activity intensity. Responses are rated on a 5-point Likert scale (1 indicating very low activity and 5 indicating very high activity). The final score is the mean of all items, ranging from 1 to 5, with higher scores reflecting greater levels of physical activity. The questionnaire is user-friendly and time-efficient, typically requiring 5–10 min to complete. It is well-suited for use in school health assessments, adolescent obesity intervention studies, and evaluations of physical activity promotion initiatives. Previous studies have shown that the Chinese PAQ-A is reliable and valid, with the Cronbach’s α between 0.70 and 0.85, a good test-retest reliability (Intraclass Correlation Coefficient, *ICC* > 0.75), and a moderate link with actual measurement results (*r* = 0.3–0.5) (Regier et al., 2013).

### 2.3 Statistical analysis

#### 2.3.1 Risk factors of head forward posture

R software (version 4.1.2, R Studio, USA) was used to analyze risk factors. For continuous data, the mean and standard deviation (SD) were utilized to describe the data. Variance homogeneity was assessed using Levene’s test, and data normality was verified using the Shapiro-Wilk test. Student’s t-test was applied for normally distributed data with homogeneous variance; otherwise, Welch’s *t*-test was adopted. For categorical variables, frequencies (*n*) and percentages (%) were calculated, and comparisons between groups were conducted using the chi-square test. Univariate logistic regression models evaluated associations between forward head posture and individual risk factors, calculating regression coefficients (*β*), odds ratios (OR), and corresponding 95% confidence intervals (95% CI). Age and daily physical activity were included as covariates for adjustments. To ensure comparability of the regression results, continuous data were standardized as follows: (value - mean)/standard deviation. All regression results were reported per 1-standard-deviation increase. Statistical significance was established using two-tailed tests, with *α* set at 0.05.

The dataset was randomly partitioned into training (70%) and validation (30%) subsets using a stratified random sampling approach provided by the “caret” package in R software (version 4.1.2, R Studio, USA). Participants with incomplete or erroneous data were excluded from the analysis. Specifically, the “createDataPartition” function from the “caret” package was utilized to ensure proportional representation of forward head posture status across both datasets, maintaining the 7:3 distribution ratio. This stratified approach guarantees balanced distribution and reproducibility, ensured by setting a random seed using the “set.seed” function. Ultimately, this method yielded no significant differences in prevalence rates of forward head posture between training and validation datasets (Kuhn, 2008; Kuhn, 2013).

Variable selection was conducted using the Least Absolute Shrinkage and Selection Operator (LASSO) method, implemented through the “glmnet” package in R (Yuan et al., 2012). LASSO performs both variable selection and regularization, effectively mitigating multicollinearity by shrinking certain regression coefficients to zero. Consequently, redundant predictors are excluded, enhancing model stability and interpretability. Although certain variables, such as BMI, are derived from other variables like height and bodyweight, LASSO assesses the individual predictive contributions of each independently, potentially retaining unique risk-related variables.

Initially, forward head posture was defined as a binary outcome variable. LASSO regression was applied to all candidate variables within the training dataset, employing 10-fold cross-validation to determine the optimal penalty parameter (lambda, *λ*). Input variables were centered and scaled during this process. The selected predictors with non-zero coefficients at the optimal lambda were subsequently incorporated into a multivariable logistic regression model to construct the final predictive nomogram using the “rms” package (Wang et al., 2018). The nomogram visually translates logistic regression coefficients into a clinically interpretable risk-prediction tool, allowing clinicians to easily calculate individual probabilities of forward head posture.

#### 2.3.2 Selection of machine learning predictive model and explanation

In the training and validation sets, 6 machine learning models were built using feature variables to predict forward head posture in primary school children: K-nearest neighbor (KNN), light gradient boosting machine (LGBM), extreme gradient boosting (XGBoost), random forest (RF), logistic multivariate regression (LM), and support vector machine (SVM). 10-fold cross-validation was used to find the best parameters for each model. Independent validation was then performed on the test set. Receiver operating characteristic (ROC) curves were drawn to evaluate how well each model could distinguish outcomes. The SHAP algorithm was used to explain the best-performing model, helping to make the model easier to understand and more transparent.

## 3 Results

### 3.1 Baseline characteristics

After screening, 514 out of 520 primary school children were included in the study dataset. Among them, 300 had forward head posture (58.37%). All participants were randomly divided into a training set (*n* = 360) and a validation set (*n* = 154) using a 7:3 ratio. As shown in Table 2, children with forward head posture had significantly higher age, height, bodyweight, BMI, average daily homework time, average weekly homework time, and ear-shoulder distance than those without forward head posture (*P* < 0.001).

**TABLE 2:** Baseline information.

Item	Total (*n* = 514)	Without forward head posture (*n* = 214)	Forward head posture (*n* = 300)	Variance homogeneity *P*-value	*P*-value
Sex					0.154
Male	442 (85.99%)	178 (83.18%)	264 (88.00%)		
Female	72 (14.01%)	36 (16.82%)	36 (12.00%)		
Age (year)	9.00 ± 2.00	8.00 ± 1.50	10.00 ± 1.00	0.03	<0.001*
Height (cm)	137.00 ± 11.50	131.00 ± 11.00	141.00 ± 8.50	0.751	<0.001
Bodyweight (kg)	30.70 ± 9.80	27.90 ± 6.58	34.65 ± 7.15	0.153	<0.001
BMI (kg/m^2^)	16.59 ± 1.50	15.69 ± 1.12	16.94 ± 1.34	0.093	<0.001
Body-type					0.058
Very thin	4 (0.78%)	2 (0.94%)	2 (0.67%)		
Thin	12 (2.34%)	6 (2.83%)	6 (2.00%)		
Normal	354 (69.14%)	148 (69.81%)	206 (68.67%)		
Overweight	92 (17.97%)	44 (20.75%)	48 (16.00%)		
Obesity	50 (9.77%)	12 (5.66%)	38 (12.67%)		
Daily homework time (hours)	1.43 ± 1.07	1.29 ± 1.10	1.54 ± 1.02	0.125	<0.001
Weekly homework time (hours)	8.41 ± 7.13	7.47 ± 7.17	9.09 ± 6.85	0.115	<0.001
Ear-to-shoulder distance (cm)	1.60 ± 1.20	0.30 ± 0.30	3.20 ± 1.10	<0.001	<0.001*

*: A variance test P-value >0.05 means variances are not equal, so Welch’s *t*-test is used to compare groups.

### 3.2 Risk factors of forward head posture

**FIGURE 3:**
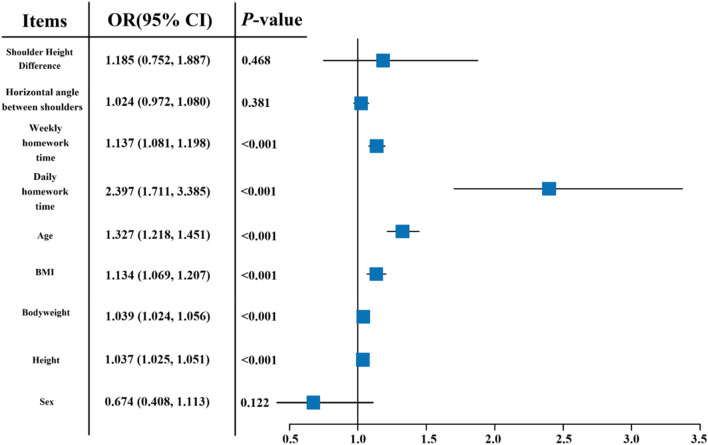
Forest plots of single-variable logistic regression for risk of head forward posture.

The univariate logistic regression results for risk factors of forward head posture in primary school children are shown in Figure 3. Age, height, bodyweight, BMI, average daily homework time, and average weekly homework time were all statistically significant (*P* < 0.001). Their odds ratios (95% CI) were 1.318 (1.189, 1.468), 1.035 (1.02, 1.051), 1.041 (1.022, 1.061), 1.161 (1.078, 1.256), 1.123 (1.056, 1.195), and 2.188 (1.46, 3.309), and they can be used as predictors for forward head posture in children.

**FIGURE 4:**
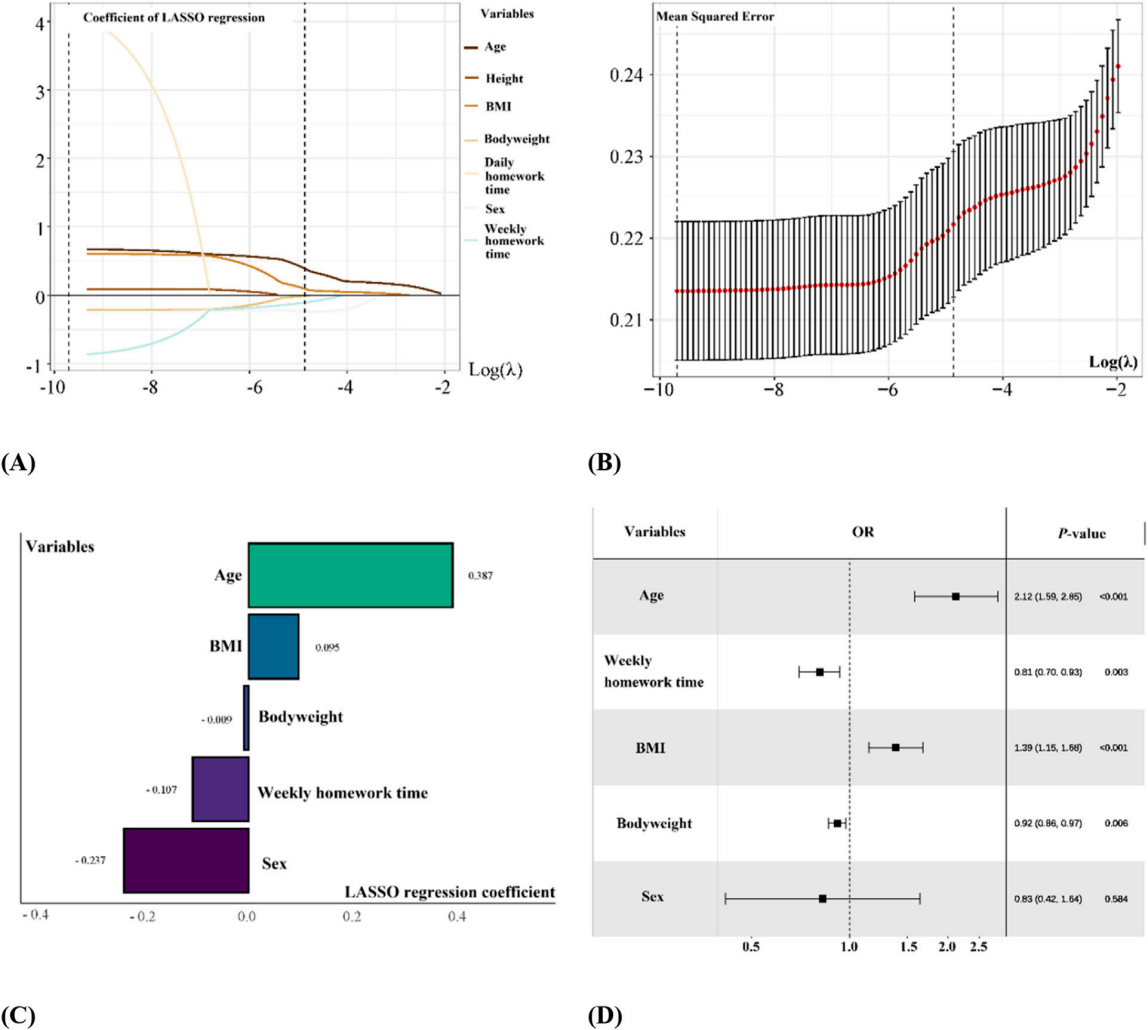
LASSO regression for feature variables selection and multi-variable logistic regression model establishment. **(A)** gg-plot of Log(*λ*) of LASSO regression for variable selection and optimization; **(B)** gg-plot of 10-fold cross validation; **(C)** Feature variables selected by LASSO regression with regression coefficients; **(D)** Forest plots of feature variables selected by LASSO regression.

Figure 4 shows the results of forward head posture determination for all participants. Figure 4A shows the feature selection and tuning process using the LASSO algorithm. Figure 4B shows the results of 10-fold cross-validation. Figure 4C shows the variables kept by LASSO and their corresponding multivariable regression coefficients. The results showed that for forward head posture, the features selected by the LASSO algorithm were age, bodyweight, BMI, sex, and average weekly homework time. Their multivariable regression coefficients were 0.387, −0.009, 0.095, −0.237, and −0.107, respectively. Figure 4D shows the forest plot of the multivariable logistic regression model built using the features selected by the LASSO algorithm.

Table 3 is a combined table of univariate and multivariate logistic regression models built from both the original features and the features selected by the LASSO algorithm. According to the table, the odds ratio (OR) means for “age” increased from 1.32 to 2.12 after being included by LASSO. The OR mean for “average weekly homework time” decreased from 1.12 to 0.809. The OR mean for “BMI” increased from 1.16 to 1.385. The OR mean for “bodyweight” decreased from 1.04 to 0.916. The OR mean for “sex” increased from 0.57 to 0.826. Moreover, among the variables found to be statistically significant (*P* < 0.05) in the univariate logistic regression analysis, “average daily homework time” and “height” were not retained in the final model selected by the LASSO algorithm.

**TABLE 3:** Single-to multi-variable logistic regression results.

Variable	Uni-variate logistic regression					Multi-variate logistic regression				
	Coefficient	SE	OR (95% CI)	*Z*-value	*P*-value	Coefficient	SE	OR (95% CI)	*Z*-value	*P*-value
Age	0.276	0.05	1.32 (1.19, 1.47)	5.15	<0.001	0.749	0.15	2.12 (1.59, 2.85)	5.02	<0.001
Shoulder height difference	0.053	0.28	1.06 (0.61, 1.85)	0.19	0.85					
Horizontal angle of shouders	0.001	0.03	1.00 (0.94, 1.07)	0.04	0.968					
Weekly homework time	0.116	0.03	1.12 (1.06, 1.20)	3.68	<0.001	−0.211	0.07	0.809 (0.701, 0.930)	−2.94	0.003
Daily homework time	0.783	0.21	2.19 (1.46, 3.31)	3.76	<0.001					
BMI	0.149	0.04	1.16 (1.08, 1.26)	3.84	<0.001	0.326	0.10	1.385 (1.147, 1.684)	3.34	0.001
Bodyweight	0.04	0.01	1.04 (1.02, 1.06)	4.12	<0.001	−0.088	0.03	0.916 (0.860, 0.974)	−2.78	0.006
Height	0.034	0.01	1.04 (1.02, 1.05)	4.48	<0.001					
Sex	−0.563	0.32	0.57 (0.30, 1.07)	−1.74	0.082	−0.191	0.35	0.826 (0.416, 1.642)	−0.55	0.584

**FIGURE 5:**
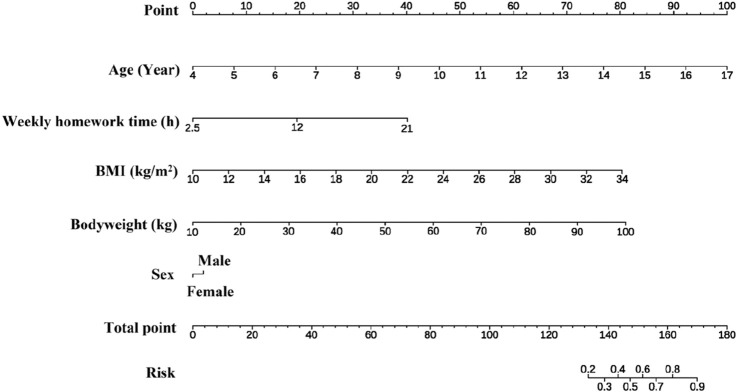
Nomogram of head forward posture prediction.

Figure 5 illustrates the nomogram derived from our multivariable logistic regression model, incorporating all key predictors identified for outcome prediction (e.g., Patient Age, Biomarker X level, Presence of Condition Y, etc.). Each predictor is displayed as a horizontal axis with tick marks indicating its range of values, and the top row of the nomogram corresponds to a Points scale. For any given value of a predictor, one can read upwards to the Points scale to determine how many points that value contributes to the overall risk score, reflecting the variable’s relative influence in the model.

To use the nomogram for an individual patient, the clinician locates the patient’s value on each predictor’s axis and draws a vertical line up to the Points scale to record the points for that predictor. After repeating this for all predictors, the points are summed and the total is marked on the Total Points axis. Finally, by drawing a straight line downward from the total points value to the bottom Risk Probability line, the corresponding estimated probability of the outcome is obtained. In this way, Figure 5 provides an intuitive graphical tool: the nomogram’s structure enables easy calculation of a patient’s risk by visualizing the contribution of each variable, and the total points translate directly into an individualized predicted risk. This explicit representation of variables and their meanings makes it straightforward to interpret how each factor influences the prediction.

### 3.3 Selection of machine learning predictive model and explanation

#### 3.3.1 Predictive performance

**FIGURE 6:**
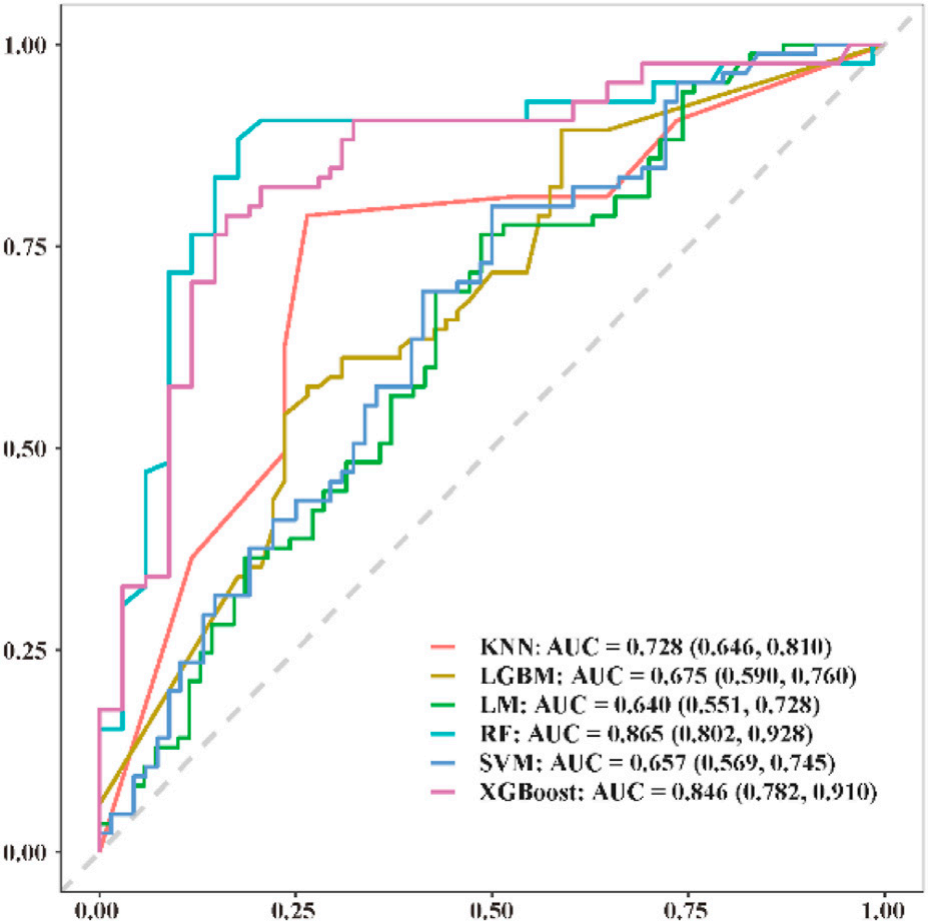
Comparison of ROC curves within difference machine learning predictive models.

The predictive performance of the 6 models was assessed using the validation dataset, and the comparative results of the ROC curves for each machine learning model are presented in Figure 6. The ROC analysis demonstrated that, compared to the traditional logistic multivariate regression model (LM) with an AUC of 0.640, the random forest (RF) model exhibited superior discriminatory power and predictive performance, achieving a notably higher AUC of 0.865.

#### 3.3.2 Model explanation

**FIGURE 7:**
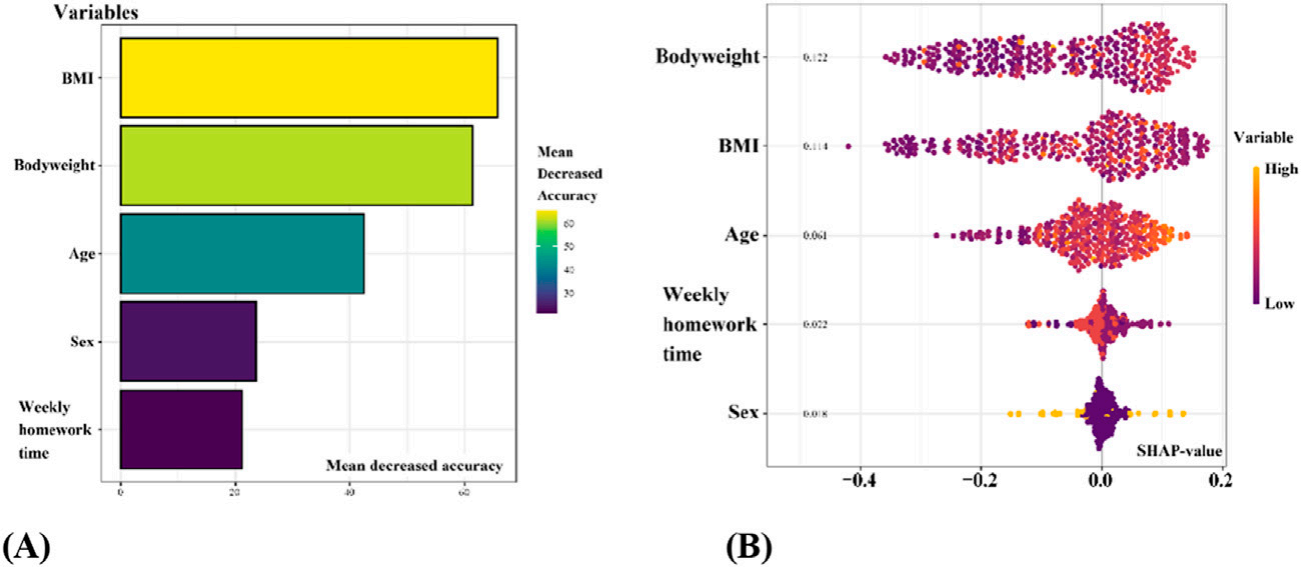
Summary of feature variables importance distribution in random forest predictive model for head forward posture. **(A)** Feature variables importance distribution plot based on mean absolute SHAP values; **(B)** feature variables distribution plot based on SHAP values.

Figure 7A is a bar chart showing the importance of features in the RF model, ranked by the average absolute SHAP values from highest to lowest. It shows how much each feature affects the model’s prediction. The vertical axis lists the features in order of importance. The results show the following order of importance: BMI > bodyweight > age > gender >average weekly homework time. Figure 7B displays the SHAP value distribution for the features in the RF model. The vertical axis lists clinical features ranked by importance, while the horizontal axis indicates the corresponding SHAP values. The color gradient of each point represents the magnitude of the feature value, allowing for interpretation of how each variable contributes to the risk of forward head posture in primary school children.

## 4 Discussion

The key findings of this study are as follows. First, primary school children with forward head posture exhibited significantly higher values in age, height, bodyweight, body mass index (BMI), average daily and weekly homework duration, and ear-to-shoulder distance compared to those without forward head posture (*P* < 0.001). Univariate logistic regression analysis identified age, height, bodyweight, BMI, and both daily and weekly homework duration as significant predictive variables for forward head posture (*P* < 0.001). Additionally, the LASSO algorithm selected age, bodyweight, BMI, sex, and weekly homework time as the most relevant features. Lastly, a comparison of different machine learning models demonstrated that the random forest (RF) model (AUC = 0.865) outperformed the traditional multivariate logistic regression model (AUC = 0.640) in predictive performance. According to the Shapley Additive Explanations (SHAP) analysis, the ranking of feature importance in the model was: BMI > bodyweight > age > sex >average weekly homework time.

This study found that children with forward head posture had higher age, height, bodyweight, and BMI, which may be linked to changes in load on the neck and muscles around it (Kwon et al., 2015; Gadotti et al., 2005). As children grow older, their bodies develop quickly, including bones, muscles, and joints. As they grow taller, their center of gravity rises, especially the head being higher than the rest of the body. This puts more pressure on the neck and shoulder muscles, which must work harder to support and stay stable. Studies show that during growth spurts, the load on the neck increases, and holding poor posture for a long time can easily lead to forward head posture (Caneiro et al., 2010). Higher bodyweight and BMI also affect posture by putting too much pressure on the neck. Over time, neck muscles get tired and cannot keep the head in the right position, which leads to forward head posture. If the child does not get enough exercise, the muscles cannot recover well, and the problem may get worse. Studies have shown that overweight children have more pressure on their neck and lower back, making it harder to sit or stand still, and forward head posture is more common (Maciałczyk-Paprocka et al., 2017; Molina-García et al., 2020). Also, taller children face more stress on the neck, especially when furniture is not ergonomically suitable or due to habitual poor posture while using devices (Hellsing et al., 1987; Mcevoy and Grimmer, 2005). Thus, early interventions addressing biomechanical stress are crucial for preventing forward head posture.

Among lifestyle and physical activity-related factors, prolonged homework duration significantly correlates with forward head posture, likely due to prolonged sedentary behavior and inadequate physical activity. Extended periods of sitting place sustained tension on neck, shoulder, and back muscles, exacerbating muscular fatigue and weakness, ultimately contributing to forward head posture (Hong et al., 2008; Baradaran Mahdavi et al., 2022). Reduced physical activity further decreases muscle strength and resilience, amplifying the negative effects of sustained static postures (Dejanović et al., 2015; Kim et al., 2008). The relationship between prolonged sedentary behaviors and poor posture underscores the importance of integrating regular physical activity into children’s daily routines (Côrrea and Bérzin, 2007).

After LASSO selection, some predictors significant in univariate analysis—such as height and daily homework time—were removed due to multicollinearity with retained variables like age and weekly homework duration. This statistical adjustment enhances model stability by retaining only independently significant variables, thereby improving interpretability (Lijin et al., 2020).

From a biomechanical perspective, modeling forward head posture based on age, bodyweight, BMI, sex, and weekly homework duration is both logical and advantageous. These factors directly influence the biomechanical integrity of posture. Increased bodyweight and BMI lead to higher cervical loading and potential muscle fatigue, contributing to postural deviations (Calcaterra et al., 2022; Babydov et al., 2023). Sex differences in musculoskeletal development may also influence posture stability, highlighting the biomechanical relevance of these predictors (Babydov et al., 2023). Moreover, prolonged sitting associated with homework contributes significantly to sustained muscular strain, reinforcing the biomechanical rationale for their inclusion as predictors (Beale et al., 2020; Davis et al., 2009).

Comparative analysis of various machine learning algorithms indicated that the random forest (RF) model provided superior predictive accuracy (AUC = 0.865) compared to other methods tested. RF effectively captures complex, non-linear interactions among multiple variables, thus offering robust predictive capabilities particularly suitable for multifactorial biomechanical conditions like forward head posture (Rigatti, 2017; Zhang et al., 2020; Roy and Larocque, 2012). The SHAP analysis further clarified the individual contributions of each variable, enhancing the interpretability of the RF model results (Bogdanis, 2012).

BMI emerged as the most critical predictor according to SHAP analysis, highlighting its dual role as a direct biomechanical factor and as an indicator of lifestyle-related behaviors such as sedentary habits and physical inactivity. Elevated BMI levels significantly increase cervical musculature strain, thus providing a crucial target for clinical interventions aimed at improving posture (Hong et al., 2008; Bogdanis, 2012).

The nomogram developed from multivariate logistic regression enhances clinical applicability by translating complex model predictions into a user-friendly visual format. This graphical tool allows practitioners to assess individual patient risk easily and aids in communication and decision-making. While the nomogram facilitates clinical interpretability, the RF model complements this by maximizing prediction accuracy through complex, non-linear modeling. Integrating the nomogram and RF model thus creates a balanced approach, ensuring both ease of use and robust predictive performance.

This study has limitations, including a relatively small sample size which may restrict generalizability. Additionally, the dataset did not comprehensively incorporate socioeconomic or additional lifestyle factors like ergonomics or detailed screen time habits, potentially limiting predictive scope. Although LASSO effectively streamlined variable selection, relevant variables could still have been excluded inadvertently. While the RF model achieved robust predictive performance, its interpretability is inherently limited. Future research should explore deep learning models, such as convolutional neural networks (CNN) or recurrent neural networks (RNN), and incorporate broader datasets to further improve predictive accuracy and practical clinical utility.

## 5 Conclusion

This study successfully developed a predictive model for forward head posture risk among primary school-aged children by utilizing the LASSO algorithm in combination with multiple machine learning model comparisons. The LASSO algorithm selected age, bodyweight, BMI, gender, and weekly homework time as key features, and the Random Forest (RF) model built using them performed the best. Among all key features, BMI was the most important predictor, showing greater influence than other variables, which highlights the importance of bodyweight control and physical activity for young children.

## Data Availability

The original contributions presented in the study are included in the article/Supplementary Material, further inquiries can be directed to the corresponding authors.
